# Consultant psychiatrists’ experiences of and attitudes towards shared decision making in antipsychotic prescribing, a qualitative study

**DOI:** 10.1186/1471-244X-14-127

**Published:** 2014-05-01

**Authors:** Andrew Shepherd, Oliver Shorthouse, Linda Gask

**Affiliations:** 1 University of Manchester, Manchester, UK

**Keywords:** Antipsychotic prescribing, Shared decision making, Patient centred medicine

## Abstract

**Background:**

Shared decision making represents a clinical consultation model where both clinician and service user are conceptualised as experts; information is shared bilaterally and joint treatment decisions are reached. Little previous research has been conducted to assess experience of this model in psychiatric practice. The current project therefore sought to explore the attitudes and experiences of consultant psychiatrists relating to shared decision making in the prescribing of antipsychotic medications.

**Methods:**

A qualitative research design allowed the experiences and beliefs of participants in relation to shared decision making to be elicited. Purposive sampling was used to recruit participants from a range of clinical backgrounds and with varying length of clinical experience. A semi-structured interview schedule was utilised and was adapted in subsequent interviews to reflect emergent themes.

Data analysis was completed in parallel with interviews in order to guide interview topics and to inform recruitment. A directed analysis method was utilised for interview analysis with themes identified being fitted to a framework identified from the research literature as applicable to the practice of shared decision making. Examples of themes contradictory to, or not adequately explained by, the framework were sought.

**Results:**

A total of 26 consultant psychiatrists were interviewed. Participants expressed support for the shared decision making model, but also acknowledged that it was necessary to be flexible as the clinical situation dictated. A number of potential barriers to the process were perceived however: The commonest barrier was the clinician’s beliefs regarding the service users’ insight into their mental disorder, presented in some cases as an absolute barrier to shared decision making. In addition factors external to the clinician - service user relationship were identified as impacting on the decision making process, including; environmental factors, financial constraints as well as societal perceptions of mental disorder in general and antipsychotic medication in particular.

**Conclusions:**

This project has allowed identification of potential barriers to shared decision making in psychiatric practice. Further work is necessary to observe the decision making process in clinical practice and also to identify means in which the identified barriers, in particular ‘lack of insight’, may be more effectively managed.

## Background

In an address to the Royal College of General Practitioners Balint described her thoughts relating to a different approach to the nature of the doctor-patient relationship [1]. She described two different forms of medicine; one in which the illness or disease lies at the centre of care and all efforts are directed towards isolating the cause of disorder and its treatment. Her alternative suggestion was that the practitioner place the patient at the centre of their thoughts, seeking to understand their beliefs and aspirations, thus adopting a more holistic approach.

“…*there is another way of medical thinking which we call 'patient-centred medicine'. Here, in addition to trying to discover a localizable illness or illnesses, the doctor also has to examine the whole person in order to form what we call an 'overall diagnosis'.*”

The sentiment was not completely novel with variations having been recognised in antiquity; Hippocrates urged the physician to make the patient their first concern.

The modern evolution of this concept is recognised as “Patient centred medicine” [2]. At the heart of patient centred medicine is the necessity for shared decision making. Varying models of the physician-patient dyad have been proposed, with differing degrees of power placed with each participant. At one pole of these lies the paternalistic model, wherein all power and responsibility lies with the clinician. The opposite model sees the patient as expert “user”, with the clinician acting solely as a source of technical information and all decision making capacity lying with the patient, the so called informed decision making model. The shared decision making model can be conceptualised as occupying the middle ground between these two poles [3]. Shared decision making has been referred to as the “pinnacle of patient centred medicine” and has become a major focus of research attention, particularly in primary care settings [4,5].

Within mental health settings the concept has been less thoroughly explored [6,7]. However the approach can be seen as being central to the personal recovery approach [8]. Empowered “use”, instead of passive “taking” of medication has been described by individual service users in their description of personal recovery [9]. Increasing individual autonomy, empowerment, and sensitive introduction of hope may be central to a shared decision making process and are well recognised themes within personal recovery narratives [10]. Despite service user preferences for greater involvement in decisions relating to their care barriers to shared decision making within psychiatric practice have been described [11,12]. Additionally it has been recognised that the discussion between psychiatrist and service user in relation to medication is open to the potential for coercion, with pressure being exerted to ensure the individual remains “compliant” with their prescribed medication regime [13].

Seeking to clarify the meaning of shared decision making, Charles and colleagues outlined a theoretical framework of the physician-patient interaction [3]. This initial formulation was subsequently revised in order to recognise the complexity of the interaction [14]. Their second model provided detail of stages involved within the decision making process as well as recognition that the process may be more dynamic than originally recognised, with clinicians sometimes needing to take on more or less responsibility as the patient demands. The framework outlines three key analytical stages in the process; information exchange, deliberation and decision. Within the theme of information exchange it was recognised that the nature of the process would vary depending on the model adopted, descriptors were therefore added to the framework to describe the nature of information flow (unilateral or bilateral), direction of transfer (physician to patient or patient to physician), information type (medical or personal) and volume (minimal or full). A paternalistic model would therefore be characterised by unilateral information flow from physician to patient with only medical information being transferred in the minimum amount possible, deliberation and ultimate decision making would lie solely with the physician. An informed patient model would be characterised by similar information transfer, although in greater volume, but this time with the deliberation and decision making stages lying solely with the patient. Shared decision making is characterised by the bilateral flow of information, between clinician and patient, with deliberation and decision making divided between the two parties.

This project sought to build on the previous work of Seale and colleagues assessing the attitudes of consultant psychiatrists towards decision making in the process of antipsychotic prescribing [11]. Increased understanding regarding the nature of the doctor-patient relationship might allow knowledge to be developed relating to practices that enable individuals to progress in their personal recovery. Decisions relating to medication are recognised as being some of the most important to both clinicians and service users, and also one of the areas where individuals are most likely to disagree with the ultimate decision when not involved in the process [12]. Our expectation was that participants would express support in principle for the shared decision making model, however it was also anticipated that a number of barriers to full implementation of this model may be perceived. Application of the framework described by Charles and colleagues would allow these barriers to be explored. Qualitative methods of investigation were employed in order that the experiences of participating consultant psychiatrists could be adequately captured.

## Methods

### Ethical approval

This research project focussed solely on the experience of consultant psychiatrists. Participants were professionals recruited on the basis of their clinical role and no confidential information relating to service user care was discussed. As such no research ethics committee approval was required in line with guidance from the National Research Ethics service.

### Participant recruitment and consent

Participants were recruited from within two NHS Mental Health Foundation Trusts situated within Manchester and the surrounding area. Participant recruitment and data analysis were conducted in parallel such that subsequent rounds of recruitment were theoretically driven with participants being selected to address emergent themes from the preceding interviews, broaden the scope of clinical experience and to seek contradictory evidence for emergent themes.

Participants were therefore sought from a variety of clinical backgrounds with varying lengths of experience practicing as a consultant psychiatrist. No specific inclusion criteria were specified excepting that the participant currently hold a post working as a consultant psychiatrist. Participants working specifically in Child and Adolescent, Later Life or Learning Disability fields were not sought as it was believed that the specific requirements of their work may restrict the applicability of their experience to the research question, by restricting choice of antipsychotic medication for example.

Potential participants were initially approached through electronic communication, with an explanation of the goals of the project being circulated. If an interest in participation was expressed an appointment of one hour was scheduled. During this meeting the purpose and methods of the project were repeated and participants were asked to consent to participate in the study. Participants provided written informed consent for participation after opportunity was provided for discussion of the aims of the research and the methods to be employed. Explanation was provided that efforts would be made during the transcription process to ensure participant anonymity. Opportunity was offered for withdrawal from the study at any point in the process, including following completion of the interview on review of the completed transcript.

On completing the interview participants were asked to suggest a potential interview candidate that they would approach were they conducting a similar study, particularly someone who may describe views or experiences different to their own.

Participant recruitment continued until data saturation was reached with no additional themes emerging from subsequent interviews.

### Interview structure and collection

Interviews were conducted according to a semi-structured interview schedule specifying topics to be specifically covered during each interview, with probe questions to elicit attitudes towards shared decision making practices. Initial topics addressed included clinical experience, factors influencing the choice of antipsychotic prescription, involvement of the service-user in the decision, the role of other professionals in the decision and a request for a recent illustrative clinical case example of the principles involved. However the structure was not rigidly adhered to, with participants being invited to draw on clinical cases from their own experience to illustrate their discussion. The interview schedule was adapted following each interview in order that newly emerging themes could be adequately addressed in subsequent interviews. Additionally participants were invited to suggest one question that they would like addressed in future interviews, these proposed questions were included in subsequent interviews.

Interviews were conducted at the participants place of work, generally within a private office space. All interviews were conducted on a one-to-one basis. Interviews were recorded using a digital-audio device. The duration of all interviews was approximately one hour. Interviews were transcribed verbatim from audio recordings. Completed transcripts were reviewed to ensure that complete participant anonymity was maintained. Transcripts of the completed interviews were forwarded to the respective participants for comment.

### Data analysis

Interview transcripts were analysed in a line by line fashion, with sections of text being highlighted in relation to their description of the decision making process and involvement of patients in this process. Sections of text thus identified were coded according to their description of the process; for example in relation to information sharing, decision making or external influences on the process. Comparison of themes between transcripts was made with supporting and contradictory evidence being sought.

Transcript coding and participant recruitment were discussed in supervision with LG with suggestions being made for alternative coding strategies and subsequent focused recruitment to address emergent themes.

As described above participant recruitment continued until data saturation was reached, this was judged to be the case when no new themes emerged from two subsequent interviews.

Themes identified from analysis of transcripts were subsequently compared with the framework of shared decision making described by Charles and colleagues [14]. Coding was therefore completed in relation to the processes described in the framework with supporting and contradictory themes sought. Themes not represented adequately by the framework were also sought. The overall analysis strategy was therefore consistent with a directed analysis method [15].

Overall the methods and ethics approval employed within this study are consistent with qualitative research review guidelines (RATS).

## Results and Discussion

### Research Participants

A total of 27 consultant psychiatrists responded to electronic communication and were approached directly, one of whom refused consent to participate. This participant stated they did not feel there was a research question to be answered in the area proposed for discussion and therefore declined to take part. After interview on receiving the completed interview transcript one participant requested that an alteration be made, which served to clarify, not alter, the meaning of the content. Participants were selected from a variety of clinical backgrounds including; Inpatient general adult, community general adult, medical liaison psychiatry, forensic psychiatry and medical psychotherapy. Length of clinical experience working as a consultant psychiatrist varied from three months to 31 years. The mean length of consultant practice was 13 years, there was a bias within the sample towards more experienced clinicians which emerged as a result of the purposive sampling strategy.

### Directed analysis and shared decision making framework

Themes are described below in relation to the shared decision making process according to the framework outlined by Charles and colleagues [14]. Illustrative quotes are presented from interviews, together with the participant code. Where possible contradictory quotes are also presented. The number of participants describing factors relating to each of the framework headings are presented - these numbers need to be interpreted with caution however as the sampling methods used, in keeping with a qualitative approach to understanding, prevent generalisations being drawn in terms of the representativeness of numbers.

Most participants presented the choice of antipsychotic decision making process as being one that should be shared between the psychiatrist and service user:

“*Well the first thing to say of course is that you don’t just make that decision yourself; that decision is a joint decision between you and the patient, because you might put them on what you think is the best anti psychotic, but they might be unwilling to take that medication for whatever reason, side effects or anything else*.” [Int023]

“*[I]f you can maximise peoples’ autonomy, we’re all supposed to be in favour of autonomy, so, you know, using a sort of Beauchamp and Childress view, I’d be quite happy for that, as a good, in itself, as an outcome.*” [Int015]

Respect for the individual’s autonomy represents one of the four principles of medical ethics outlined by Beauchamp and Childress in their Principles of Biomedical Ethics [16].

The expression of support for shared decision making voiced by participants in this study is not in keeping with observations of psychiatric clinical practice however, where consultant psychiatrists were found to perform poorly on measures designed to assess the involvement of service-users in clinical decisions [17,18]. Similarly research exploring the nature of shared decision making consultations has highlighted the prevalence of *scientific based* discussions, relating to relative merit of treatment options, in relation to individual *preference based* discussion where alternative treatment options are considered [19].

In discussing the initiation of antipsychotic therapy some participants recommended a period of medication-free assessment before prescribing. Only one participant proposed that there may be an alternative to antipsychotic prescribing as the primary method of treating psychosis - this view was presented by a Medical Psychotherapist, who also utilised prescribing as part of their clinical practice, they proposed that psychotherapy be used as the treatment of choice with antipsychotic medication being used as an adjunct only if necessary.

*“…I think, I don’t, its my personal bias isn’t it? That, that, I would have therapy and see that as the primary way of working through.”* [Int014]

Views relating to the primacy of antipsychotic medication as the technological treatment modality for psychosis will likely influence the manner in which information is presented to patients during consultation.

In common with the updated framework described by Charles and colleagues one participant described the process of involving service users in decision making as particularly complex, necessitating a change in role for the clinician, or shift in responsibility for decisions dependent on the context.

“*You need, I think, as a consultant psychiatrist to be able to fit into both roles, to be able to be a, sort of, educated supervisor and advisor for a family with an illness that they themselves can manage and you can support them with and then you need to be somebody who can take all the clinical decisions away from somebody when necessary when somebody is very, very acutely unwell.*” [Int003]

Having expressed their support for the principle of shared decision making as a model of care participants provided further details on the process as they understood it within their own practice, including perceived limitations. Table 1 provides a summary of the emergent themes in relation to their respective framework heading - detailed discussion of themes and their ability to support or frustrate the shared decision making process are provided below.

**Table 1:** Summary of themes relating to framework headings

**Information sharing**	**Deliberation**	**Deciding on treatment options**
Eliciting service user preference	Engagement and discussion	Methods of information provision
Improving compliance	The role of insight	Manner of information delivery
Clinician prescribing bias	*External factors influencing clinician*	Revisiting treatment decisions
	1. Nature of the clinical environment	
	2. Pressures to discharge	
	3. Other clinical staff	
	4. Financial pressure	
	*External factors influencing the client*	
	*1.* Expectations and beliefs	
	2. The role of Mental Health Law	

#### 1. Information sharing

Eight participants described in detail in the process and nature of information sharing between clinician and patient. Participants highlighted the importance of eliciting the preferences of service users in relation to medication side-effects and incorporating this into the decision making process:

“*[I]t is something that I talk to people, or counsel people, about and if they have a strong volition, not to have a particular side-effect, or are wary, whether I think that’s a real, whether I think there’s proper evidence for that or not, you try and work with people I think.*” [Int004]

“*[T]he person in front of you will come with their own beliefs, their own wishes.*” [Int005]

Some participants described the transfer of information as being necessary to improve compliance, or as an essential process in informing another of a decision that had already been made. The term “compliance” is used advisedly in this context to represent the meaning of “taking” of prescribed medication, a passive action; this is opposed to the active act of “concordance” where the recipient recognises and fully “accepts” the role of medication [20]:

“*I tend to be very direct with patients because it increases the likelihood of taking it if you know what it’s for and what is likely to happen to you and why you’re being given it.*” [Int001]

“*I think if they do feel that they have some choice, then that is helpful, if they have a choice around, not perhaps taking medication or not, but actually some choice within the medications that they take.*” [Int018]

Previous work in this area has identified that clinicians are at times reluctant to discuss side-effects relating to a medication, believing that it may impact on the chance of the service user’s compliance [11]. This was not described during these interviews. There was a sense in which the clinician’s own perspective on side-effects might impact on their information sharing practice. Older first-generation, also referred to as “typical” antipsychotics, exemplified by Haloperidol, are recognised as leading to motor side-effects, including muscle spasms, dystonias, or rapid purposeless movements, dyskinesias. More modern second-generation, “atypical”, agents, exemplified by Olanzapine, predispose more to metabolic side-effects such as weight gain and impaired glucose tolerance [21-23]. During these interviews the distinction between antipsychotics predisposing to motor side-effects and metabolic side-effects was drawn, with a sense of responsibility for inducing the more immediate motor side-effects being described by participants, metabolic side-effects were presented as less visible and, while still of concern, the degree of responsibility, on the part of the prescribing clinician, was absent from descriptions.

“*[I]t’s so visible tardive dyskinesia… and also torsion dystonias I’ve seen, some horrible ones of those as well, that people need Botulinum Toxin and so forth… and it is extremely visible, so it’s a horrible thing to inflict.*..” [Int006]

It is proposed that biases within the clinician’s clinical experience, for example avoiding the visible side-effects of antipsychotics such as Haloperidol, will influence the manner in which information is shared and represent a point in the process where coercion may be unduly exerted.

#### 2. Deliberation

22 participants described the manner in which they engaged in discussion with service users, presenting their opinion regarding management options and refining this based on feedback received.

**“***[I]f their preference fits in with what I happen to think is the best one for them then it’s very straight forward, if it doesn’t then that that starts off a conversation about why what the advantages of them taking the medication they have chosen and perhaps the disadvantages of that and whether some other preparation might have more advantages or have certain advantages that they’ve not thought about, if in the end it doesn’t really matter that much what preparation it is then I tend to go with what the patients want.*” [Int026]

In considering the process of deliberation, jointly between service-user and psychiatrist, most participants described the role of insight as a factor in psychosis that would limit, or completely prevent, shared decision making. For some this was presented as a *restriction* that necessitated strategies to ensure the service-user was as involved as possible in the decision making process, or that family members or other potential carers were involved. For six participants the impact on insight on the individual’s capacity to consent was an *absolute contraindication* to shared decision making.

**“***Some patients are just too unwell to make that kind of decision, they can have no capacity at all to make that kind of decision at the time of admission, in which case we just have to go with what we feel is advisable at that time.*” [Int001]

“*[I]f I was to see someone, who was acutely psychotic, very behaviourally disturbed, poor insight, it’s obviously not going to be possible, at that particular point in time, to go through, all the side-effects of all the different antipsychotic medications, and expect a patient, at that point, to be able to, to engage in that process.*” [Int010]

“*So, I think, at that stage you, well I attempt to, discuss the issues with the patient but, to be perfectly honest, if it’s clear that they’re insightless, or that they’re not going to agree, and you get that, not only with the one-to-one conversation that you have, but also being aware of how they’re being, from the time they’re admitted to the unit. I don’t think there is much point, at that, moment in time, to get into a conflict.”* [Int013]

“*I think giving patients choice is um is ideal if you can but at best um at worst I should say it is rather idealistic you know I’ve got a patient in front of me who is floridly psychotic and totally insightless”* [Int024]

These participants presented insight in psychosis as a binary concept - present or absent. This has traditionally been accepted as an adequate model of insight, however more recent models propose that insight be considered a dynamic, multidimensional, process [24]. Reduced insight has been recognised as a barrier to seeking treatment and engagement with offered therapies and it has also been proposed that in such cases the adoption of more paternalistic approach to care is ethically appropriate; however qualitative research into the experiences of individuals suffering with psychotic disorders have also emphasised the importance of ensuring that autonomy be respected as far as possible throughout the clinical presentation [25,26]. In this study if the clinician considered the service-user unable to participate in the decision making process then strategies were described such that a decision could be reached in the best interests of the individual. In this way participants described taking steps to ascertain any prior wishes of the service-user, this enable them to act as an agent representing the other party. This model of decision making is generally recognised as being complicated by difficulties involved in the clinician gathering all relevant information relating to the decision to be made [27].

“*[I]t depends on the amount of information that you have, but I think I would go again back to taking a good history, and if you can’t take a good history from the patient from the informants around them, and sort of then choosing in terms of best evidence what you know about the patient, what you know about what they might have said in the past, advanced directives, things like that. You’ve got to actually take in all of that into account, and then decide, well, this drug would be the best, you know, in terms of outcomes that I think in the longer term not only would get the patient better but might be something that once they regain capacity, they will be willing to continue to take*.” [Int023]

In describing the processes involved in reaching a decision, 18 participants also detailed a number of factors that they perceived would impact on the process. These factors were external to the physician-patient relationship, but would affect both parties either individually or together. It can be proposed that the decision making encounter represents an example of where clinician and patient work together in order to construct an interpretation of experience - factors and expectations external to the therapeutic dyad may possibly exert an undue influence on this process.

##### External factors influencing the clinician

Factors external to the relationship but impacting on the decisions of the psychiatrist included pressures from the nature of the clinical environment and also from other colleagues, for example nursing staff, who contribute to the care of the service-user. In terms of the *environment* participants described their choice as being restricted by the nature of the environment that the service-user was within and a need to “contain” them safely within that environment.

“*Certainly looking at people in prison and, to a certain extent, causing problems on any unit, they just want the behaviour to settle, and I think… being honest, I don’t think they really care, as long as, yeah, yeah, but to be honest, I think, even if they just sleep, even if all the antipsychotic does is sedate them, you know, they’re happier with that then, having somebody, you know, shouting or screaming all night, or whatever it is*.” [Int013]

“*Because you have patients who are unwell, and you are managing them in their own home. So that puts in a different dynamic to your prescribing, because you need something that acts quickly, something that doesn’t have major side effects.”* [Int022]

Pressures were also described as limiting the flexibility for decisions to be reviewed in the future, particularly if there was a need for people to move through hospital quickly and be discharged.

“*The difficulty that we have in psychiatry, and I think in all medicine to be honest, about this sort of bed pressures and things like that, a lot of people get better on a medication that they were started on, and then people are very worried about rocking the boat. So they might put them on something in the beginning and they might think, oh I’ll later change it to another medication, but by that time the patient might be ready for discharge, or you feel oh well they’re just better now I don’t want to try anything different that’s going to make them unwell again, they’re back into hospital.*” [Int023]

Other clinical staff also were described as having expectations in terms of prescribing, necessitating the use of medication that others were perceived as having “faith” in.

“*I’ve got a good relationship with the nurses I work with and they’ve never fought me on any of them* [decisions]*, yet, I’m sure they will, but I’m sure they have done with other consultants and they’ve said; “we really think”,the one I’m thinking about was about Clopixol, Acuphase, they thought it would be very useful for a patient and the consultant didn’t. The consultant would bear the responsibility for that decision, not the nursing staff so, so you know, I think that’s a difficult game.*” [Int003]

Finally 18 clinicians also described the role of financial pressures impacting on their prescribing choices, particularly in the development of local guidelines or funding decisions negotiated with primary care trusts, who hold the budget for prescribing. Overall this restriction was perceived as limiting the options available to the service-user.

##### External factors influencing the client

In terms of external factors impacting on the service-user’s decision, eight participants described the role of *society’s expectations and widely held beliefs* relating to medication, in particular the view of antipsychotic medication as a having *tranquillising* effects. The specific role of medication in mental disorder, with medications representing coping strategies for both service users and psychiatrists, and the historical perception of psychiatry’s role in society, with psychiatrists viewed as reducing human suffering to a purely biological process, were also discussed. The ability of psychiatrists to provide treatment through use of *Mental Health Law* was also highlighted as limiting potential engagement between psychiatrist and service-user, participants felt that concern relating to the perception of Mental Health Law as allowing compulsory treatment would erode trust between the service-user and psychiatrist.

“*So, the other thing is, that the drugs are often used as transition objects, so having them makes them feel like they’ve got something that’s like a safety blanket, so they feel better because they’ve got something to take, so often they want to have drugs, even if they’re not taking them, so they want the prescription, they want to have the bottle in their hand, but then they won’t take them.*” [Int014]

“*I think a complete lay person thinks antipsychotics are some huge, heavy duty, massively sedating and tranquillising, thing that rots your brain and stops your functioning, and just sort of zombifies people. You hear people saying things like doped up to their eye balls on medication, I think that’s the perception people sometimes have, and really that’s far from [quiet laugh] necessary isn’t it.”* [Int001]

“*[T]hey would regard it as being something that is necessary to manage psychotic illnesses, I mean, I would imagine that there is a public perception of psychosis or psychotic illnesses that is, tends to be, obviously quite, negative in terms of looking at the problems people with psychosis might pose and they would concentrate really on the rare instances of risks.*” [Int002]

“*[W]hen I started, there was a perception, of you know, psychiatrists being pill pushers, you know chemical cosh, reaching for the prescription pad in the face of human distress, I’m not sure that’s changed massively*.” [Int012]

The complexities and meanings of medication within society, beyond the intended purpose on prescription, have been previously described and can be recognised as being particularly complex in the case of mental disorders where psychological and sociological processes play a substantial role [20].

#### 3. Deciding on treatment options

The role of decision support tools to support service-users and clinicians have previously been explored in relation to shared decision making [5,28]. One participant in this project also described methods of providing information to service-users.

“*With first episode people, or with patients, what we tend to is offer people a choice of four. So, we’ll talk to them about what side-effects, and effects of the medication they’re interested in, what they rate most highly is important, and then, we’ll pick four antipsychotics, that essentially offer a contrasting choice, of these different things, two first generation and two second generation, and, then we’ll let them, pick which one they want, and, and then we’ll just go down that route*.” [Int015]

Three participants highlighted the importance of optimism and presenting the choice of antipsychotic prescription not as absolute, but as a trial that could be reviewed dependent on clinical response and side-effects. This approach is consistent with that recommended in the current guidelines from the National Institute of Health and Care Excellence in the treatment of Schizophrenia [29].

“*You’ve got to give a measured approach, I try and sound optimistic, so I say, this might well work, it works for some people, but not all, what we’ve got to do is just try you on a little bit and see, and there are other things I can try, but let’s just start, one thing at a time, give it a good go, it’s up to you, and then see what happens.*” [Int011]

The concept of revisiting treatment decisions was raised by participants and was presented as being particularly problematic when decisions had been taken *without* the service-users initial involvement. A pressure was then described between the need to meet the expectations of the individual, now with capacity to engage with treatment decisions, while also maintaining on-going treatment. Clinicians acknowledged that this subsequent discussion was a time when the interaction was likely to be at risk of coercion, with pressure being exerted by clinicians to encourage the service-user to remain compliant with a treatment already commenced [13]. Prescribing decisions will also be naturally revisited in subsequent review appointments - the decision to take medication, or not, does not represent a discrete choice for the client, but is instead a continuous process. These subsequent reviews are likely therefore significant and need additional consideration.

## Conclusions

In keeping with previous research in this area participants in this study expressed support for the concept of shared decision making in antipsychotic prescribing, although as commented above this is out of keeping with observational studies of consultation practice [11]. Participants also noted however that shared decision making may not always be an appropriate model of care for individuals with a mental disorder and highlighted the need for psychiatrists to be able to recognise when the service-user wishes to take on more or less responsibility in their care. Previous studies have however also highlighted that clinicians do not always appreciate when service-users wish to be more actively involved in their care. Service-users not involved in the decision making process are more likely to disagree with the decision taken on their behalf, or to be actively resistant to the decision [12].

As previously stated this study sought to build on the existing work of Seale and colleagues [11]. In keeping with their findings participants in this study voiced support for shared decision making in antipsychotic prescribing - an observation, as described above, in contrast with service user focussed research on the same topic. Seale called for further work in different populations to further explore this finding - which our work addresses. As in Seale’s work participants in this study identified antipsychotics as the primary intervention in the treatment of Schizophrenia and related psychoses. Insight, or competence, was recognised in both studies as being a significant barrier to the sharing of decision making responsibility. Unlike in the previous study however participants herein did not describe a reluctance to impart information relating to side-effects - but did display a differential interpretation of the role of antipsychotics, and their side-effects, that possibly would impact on the nature of the information shared. By involving participants practicing in areas other than outpatient general settings our study has allowed exploration of the role of the nature of the therapeutic environment and other external influences on prescribing.

Participants in this study presented the decision making process in antipsychotic prescribing as a highly complex problem, with the ultimate decision impacted on by a number of factors external to the physician client dyad at the centre. Additionally the need for the clinician to adopt varying roles as the situation dictated was also highlighted. This complexity is represented by the revised model described by Charles and colleagues, although the specific external agencies acting on the process are likely unique to the psychiatrist-client relationship [14].

The biggest perceived intrinsic barrier to the shared decision making model proposed by participants was the concept of *insight,* which was generally considered by participants as a binary concept; no additional description of domains of insight were presented. Previous research has suggested that the conceptualisation of insight is better considered as a continuous distribution in several domains. For example a qualitative exploratory study by Greenfeld and colleagues with service users recovering from a psychotic episode identified five domains of insight - relating to symptomatology, the presence of disorder, causative factors, possibility of future relapse and the value of treatment [30]. Such concepts were developed further with the proposal of quantitative rating scales based on dimensions of insight, the relationship between degrees of insight and global level of psychopathology have subsequently been explored [31,32]. Meta-analysis demonstrated only a modest inverse relationship between degree of insight and global symptom severity, leading Mintz and colleagues to propose a possible curvilinear relationship where insight initially decreases but then increases as symptoms become more severe [32]. Such observations thus challenge the representation of insight as being merely present or absent in a binary fashion. It is not clear whether, if different dimensions of insight were more fully explored in the clinical encounter, this would assist in promoting shared decision making. Research into possible psychological techniques to foster the development of insight are underway; such techniques may be of value to support the individual and allow shared decision making in relation to proposed treatment to proceed [33].

### Limitations

A strength of this project was the inclusion of participants from a variety of clinical backgrounds which provided greater depth of information than has previously been observed as previous studies have been conducted solely within community outpatient environments. A potential limitation however is that participants were drawn solely from one region in the UK, similarity in training and clinical experience among clinicians practicing within the region may have led to significant factors being overlooked. However clinicians participating in this project completed their medical and psychiatric training in a range of areas outside the Greater Manchester area, as such this limitation is likely to have not impacted unduly on the findings.

The limitations of directed analysis as a method of qualitative data analysis must also be recognised [15]. In seeking evidence to support a proposed framework or model it is possible that contradictory examples may be overlooked, it is hoped that this possibility has been minimised in this study through the purposive sampling of participants to introduce as wide a range of views as possible. Additionally participants may have detected cues within the probing questions provided that led them to provide an answer not representative of their true behaviour or beliefs, it is hoped that encouraging the participants to describe their real-world clinical practice limits this possibility. Semi-structured interviews were conducted by OS and AS, a medical student and psychiatry trainee respectively. It is possible that the nature of the interviews may have led participants to view the process as an opportunity to provide training which may have affected their responses to questions. This limitation could only be overcome through future work involving direct, or indirect, observation of clinical encounters between these clinicians and service-users.

Data analysis was conducted by AS, under the supervision of LG. AS had previously worked with a number of the participants in a clinical capacity and it is possible that this pre-existing relationship may have impacted on interviews and data analysis. Such issues were discussed, and hopefully minimised, through supervisory meetings.

### Implications for future research

The way in which limitation of insight may impact on service users ability to participate in the decision making process requires further exploration. Particularly mechanisms through which insight maybe enhanced require attention, as well as assessment of the best manner of ensuring maximum involvement of service-users in initial decisions relating to care which can be subsequently revisited dependent on individual preference.

The apparent conflict between the *stated* intentions of psychiatrists to involve service-users in the decision making process, their reported difficulties in achieving this and previous findings that service-users are often excluded from, or minimally involved in, the process also requires further exploration. This could be achieved through analysis of meetings between service-users and psychiatrists in which these complex treatment issues are addressed. As was stated above the decision to take medication represents an on-going, continuous, process - further research to address the manner in which this fact is handled during medication review appointments is therefor necessary.

In summary participants in this study supported the principle of shared decision making in the prescribing of antipsychotics, with the caveat that the clinician would adopt a flexible position able to take more control as the situation required. The greatest perceived obstacle to shared decision making was the perception of the service user’s insight into their mental disorder, which was presented as a binary concept. This observation is in contrast to recent clinical research presenting insight as a multidimensional spectrum. Further work is required to explore more fully the nature of the clinician-client interaction and to identify means to support the shared decision making process.

## Competing interests

The authors declare that they have no competing interest.

## Authors’ contribution

All authors contributed to the initial conceptualisation of the project and drafting of the report. Interviews were completed by OS and AS. Data analysis was completed by AS with supervisory support from LG.

## Pre-publication history

The pre-publication history for this paper can be accessed here:

http://www.biomedcentral.com/1471-244X/14/127/prepub
